# Rare association of thymoma, myasthenia gravis and sarcoidosis : a case report

**DOI:** 10.1186/1752-1947-2-245

**Published:** 2008-07-25

**Authors:** Mohankumar Kurukumbi, Roger L Weir, Janaki Kalyanam, Mansoor Nasim, Annapurni Jayam-Trouth

**Affiliations:** 1 Department of Neurology, Howard University Hospital, Georgia Avenue NW, Washington, DC 20060, USA; 2 Department of Physical Medicine and Rehabilitation, Howard University Hospital, Georgia Avenue NW, Washington, DC 20060, USA; 3 Department of Pathology, Howard University Hospital, Georgia Avenue NW, Washington, DC 20060, USA

## Abstract

**Introduction:**

The association of thymoma with myasthenia gravis (MG) is well known. Thymoma with sarcoidosis however, is very rare. We presented an interesting case with coexisting thymoma, MG and sarcoidosis.

**Case presentation:**

A 59-year-old female patient with a history of sarcoidosis was admitted to the hospital with a one-day history of sudden onset of right-sided partial ptosis and diplopia. Neurosarcoidosis with cranial nerve involvement was considered, but was ruled out by the clinical findings, and MG was confirmed by the positive tensilon test, electrophysiological findings and positive acetylcholine receptor binding antibodies. On further evaluation, a CT chest scan showed a left anterior mediastinal mass and bilateral lymphadenopathy. Post surgical diagnosis confirmed the thymoma and sarcoidosis in the lymph nodes.

**Conclusion:**

When two or more diseases of undetermined origin are found together, several interesting questions are raised. It is important to first confirm the diagnoses individually. Immunologic mechanisms triggering the occurrence of these diagnoses together, are difficult to address. Although the coexistence of thymoma, MG and sarcoidosis may be coincidental, it is noteworthy to report this case because of the multiple interesting features observed as well as the rarity of occurrence.

## Introduction

The association of thymoma with myasthenia gravis (MG) is well known and amply quoted [1,2]. Thymoma with sarcoidosis however, is very rare [3]. Presented here is an interesting case with coexisting thymoma, MG and sarcoidosis.

## Case Presentation

A 59-year-old female patient was admitted to the hospital with a one-day history of sudden onset of right-sided partial ptosis and diplopia on right lateral gaze. The patient had generalized fatigue for over a year. At the age of 30, the patient had symptoms of dyspnoea and painful nodular swellings over her legs. During that time, sarcoidosis was suspected and was confirmed by lung biopsy. For the fear of adverse effects, she had declined steroid treatment. The patient had occasional flares since then until a year later, when she suffered from exacerbation of sarcoidosis in the form of polyarthritis, generalized fatigue, dyspnoea, fever and nodular swelling over legs. The patient again declined steroids and chose to remain on naproxen. Her symptoms had improved over a period of time except the generalized fatigue. She also had diabetes mellitus diagnosed at the same time, which was well controlled with metformin.

**Figure 1:**
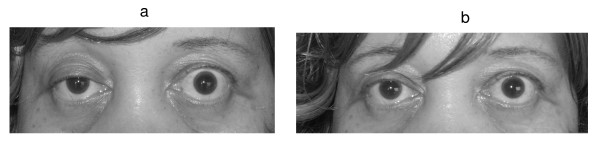
**a. Photograph of the patient showing right partial ptosis.** The left lid shows compensatory pseudo lid retraction because of equal innervation of the levator palpabrae superioris (Herring's law). b. Post tensilon test: Note the improvement in ptosis.

A neurological examination revealed a right-sided partial ptosis, and compensatory pseudo- retraction of the left eyelid (Figure 1a). Diplopia was noticed on right lateral gaze due to right lateral rectus weakness. Fluctuations in diplopia and ptosis were noticed during her hospital stay. The patient did not have any bulbar symptoms like dysphagia or dysphonia. But she was having generalized weakness in all her extremities, both proximal and distal, with marked diurnal variation in the form of more weakness during the evening.

**Figure 2:**
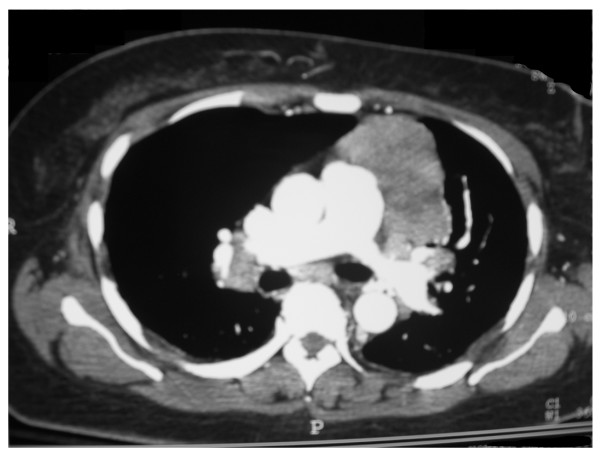
CT chest image revealing large necrotic mass in the left anterior mediastinum and bilateral hilar lymphadenopathy.

During her hospital stay she developed respiratory distress and hypoxemia. A chest CT scan demonstrated a 7 cm necrotic mass in the left anterior mediastinum and bilateral hilar lymphadenopathy (Figure 2). Pulmonary function tests revealed restrictive lung disease and moderately decreased diffusion capacity. Steroids were recommended for the active sarcoidosis, which the patient declined.

An ophthalmology consult revealed no evidence of uveitis or optic neuritis. MG was confirmed by the tensilon test that showed improvement in her ptosis (Figure 1b) and strength in muscles of the upper limb. A confirmatory electrophysiological study with repetitive nerve stimulation (RNS) showed decrement in amplitude of action potentials with further reduction post exercise and recovery after 15 minutes, consistent with MG. Acetylcholine receptor (AChR) binding antibodies were markedly elevated (104.00 nmol/L; normal < 0.30), consistent with MG.

The patient's corrected calcium level was mildly elevated (10.7 mg/dl; normal 8.5–10.4). Other relevant tests with respect to hypercalcemia were, normal renal function tests (blood urea nitrogen 18 mg/dl, normal 7–25; creatinine 0.9 mg/dl, normal 0.7–1.4; phosphorous 3.9 mg/dl, normal 2.5–4.5; magnesium 1.8 mg/dl, 1.7–2.5) and normal intact parathyroid hormone (PTH) levels (33 pg/ml, normal 10 – 69). Other tests like 1,25-dihydroxyvitamin D (OHD), 25-hydroxyvitamin D and 24 hour urinary calcium levels were not measured.

Further tests revealed a high sedimentation rate (ESR) (78 mm/hr; normal 0–30) and increased serum angiotensin converting enzyme (ACE) levels (127 u/l, normal 9–67). These results, in conjunction with the pulmonary function tests, and hilar lymphadenopathy were consistent with active sarcoidosis.

Other relevant investigations including thyroid function tests, muscle enzymes, anti nuclear antibodies, rheumatoid factor, B_12 _levels, glycosylated hemoglobin levels and rapid plasma reagin tests were normal. MRI of the brain was also normal.

**Figure 3:**
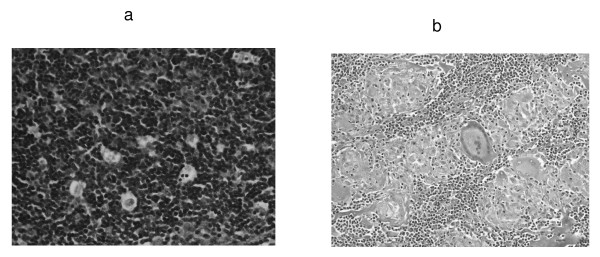
**a. Mass from the anterior mediastinum confirming thymoma B1 WHO type, lymphocyte rich predominantly cortical. (H&E stain, high magnification).** b. Biopsy from the lymph nodes showing multiple non-caseating granulomas with multinucleated giant cells and histiocytes (H&E stain, low magnification).

A CT guided fine needle biopsy of the left anterior mediastinal mass showed a predominantly lymphocytic cytokeratin positive thymoma. The patient was started on pyridostigmine with a remarkable improvement in weakness, diplopia and ptosis. Surgical removal of the thymoma with lymphadenectomy was performed. Postoperative surgical pathology demonstrated the presence of stage II A, WHO type B thymoma (Figure 3a). Lymph nodes showed noncaseating granulomas with multinucleated giant cells confirming sarcoidosis (Figure 3b).

The patient was diagnosed with having coexistent thymoma, MG and sarcoidosis.

The patient underwent post thymectomy radiotherapy. After successful counseling she was also started on a high dose of oral prednisone and oral pyridostigmine was continued. She has remained asymptomatic to date, and oral prednisone is being tapered accordingly.

## Discussion

Coexistence of thymoma, MG and sarcoidosis is very rare. A literature review revealed documentation of thymoma with sarcoidosis in two cases [3], and MG with sarcoidosis in two cases [4]. The presence of thymoma, MG and sarcoidosis together has not been discussed previously, except a single case mentioned in a MG case series without any detailed discussion [5].

Neurosarcoidosis is a complication in 5% of patients with sarcoidosis [6]. The most common neurological manifestations of sarcoidosis are cranial neuropathies from chronic basal meningitis. The facial nerve is affected most often, sometimes bilaterally. Optic nerve may also be swollen or atrophied. Ophthalmologic examination in our patient revealed normal fundus, normal 3^rd^, 4^th ^and 6^th ^cranial nerve examination and no evidence of uveitis. Other cranial nerve examinations were normal. MRI brain did not show any evidence of sarcoidosis. All these features suggested the low probability of neurosarcoidosis.

Hypercalcemia in patients with thymoma can be seen due to the following reasons, namely, secretion of parathyroid-related protein (PTHrP) or PTH by the thymoma itself, coexistence of parathyroid adenoma or hyperplasia of parathyroid gland [7]. In these cases, PTH level is usually elevated. Hypercalcemia can occur in granuloma forming disorders such as sarcoidosis, because of extra renal production of 1,25-dihydroxyvitamin D. PTH release is inhibited by hypercalcemia and high levels of calcitriol, which explains the suppressed PTH level in sarcoidosis [8]. In our patient the PTH level was inappropriately normal, which cannot exclude coexisting hyperparathyroidism. Other tests like 1,25-dihydroxyvitamin D, 25-hydroxyvitamin D, 24-hour urinary calcium levels and immunohistochemistry for PTH, would have been appropriate evaluation tests in the current case.

Approximately 30–50% of patients with thymoma have MG [1]. The symptoms of MG usually precede the discovery of a thymoma. Reports indicate the association of MG with thymoma to be about 15% but increase to 35% in older patients [2]. Ocular symptoms like diplopia and ptosis are the commonest clinical presentations in MG seen in more than 50% of patients [1]. Positive tensilon and RNS tests have sensitivities of 70 and 75% respectively [9]. AChR antibodies are elevated in 98% of patients with MG and thymoma [9]. The reported case has all these findings.

It is well known that disorders of immune response may coexist in some patients [10]. When two or more diseases of undetermined origin are found together, several interesting questions are raised. It is important to first confirm the diagnoses individually. Immunologic mechanisms triggering the occurrence of these diagnoses together, is difficult to address. Thyroid and autoimmune diseases are often observed in these cases. This patient however had normal thyroid and autoimmune profiles. Although the coexistence of thymoma, MG and sarcoidosis may be coincidental, it is noteworthy to report this case because of the multiple interesting features observed as well as the rarity of occurrence.

## Conclusion

In this case sudden onset of ptosis and diplopia was noted in a sarcoidosis patient. This could have been neurosarcoidosis with cranial nerve manifestation. But, this case revealed an unusual presentation of thymoma and MG and their rare association with sarcoidosis.

## Competing interests

The authors declare that they have no competing interest.

## Authors' contributions

MKK and RLW were involved with the management of this patient. MKK, JK and AJ–T were involved in the data collection and drafting of the manuscript. MN reviewed all pathological specimens. JK conducted the EPS study. All authors reviewed the final drafting of this manuscript.

## Consent

The authors would like to thank the patient for providing informed consent for the publication of this case report, as well as the photograph. A copy of the consent is available from the Editor-in-Chief.
